# Taking capacity seriously? Ten years of mental capacity disputes before England's Court of Protection

**DOI:** 10.1016/j.ijlp.2018.11.005

**Published:** 2019

**Authors:** Alex Ruck Keene, Nuala B. Kane, Scott Y.H. Kim, Gareth S. Owen

**Affiliations:** a Dickson Poon School of Law, King's College London, UK; b Department of Psychological Medicine, King's College London, Institute of Psychiatry, Psychology and Neuroscience, UK; c Department of Bioethics, National Institutes of Health, USA

**Keywords:** Capacity, Decision-making capacity, Mental capacity, Competence, Court of Protection, Mental Capacity Act

## Abstract

Most of the late 20th century wave of reforms in mental capacity or competence law were predicated upon the so-called ‘functional’ model of mental capacity, asking not merely whether a person had a mental disorder or disability but rather whether they were capable of making a specific decision (or decisions) at a specific point of time. This model is now under sustained challenge, most notably from the Committee on the Rights of Persons with Disabilities, and this challenge has focused a spotlight on the difficulty of applying the legally ‘neat’ concepts of the functional model of mental capacity across the full complex spectrum of human life.

This paper presents a review, in two parts, of the first ten years of the Court of Protection, a specialist mental capacity court in England and Wales which applies a functional model of mental capacity.

The first part outlines the history of the functional model in England and Wales, and the development of this specialist mental capacity court (Court of Protection), created by the Mental Capacity Act 2005. The second part presents an empirical and case-based study of 40 published cases of capacity disputes presented to the Court of Protection, or to the Court of Appeal on appeal from the Court of Protection, during the first ten years of its existence.

The authors found that in 70% of cases the subject of proceedings (or P) had either a learning disability or dementia, and the court ruled on P's capacity for a wide range of issues, most commonly residence, care and contact. The judge considered the support principle, or whether practical steps were taken to maximise P's capacity, in 23 of 40 (57.5%) cases. The subject P was determined to have capacity in 13 cases, to lack capacity in 22 cases, and in 5 cases P was found to have and lack capacity for different issues before the court. The functional inability to use or weigh relevant information was most commonly cited by the judge, being cited in all but 2 cases in which P was determined to lack capacity and inabilities were cited. The propensity for the system to learn was shown by an increase in the proportion of cases which considered the ‘causative nexus’ from 2013, when a Court of Appeal case emphasised that impairment must not merely be present alongside functional inability but must be the causal basis of inability.

The authors conclude that whilst the Court of Protection is still on a learning curve, its work provides a powerful illustration of what taking capacity seriously looks like, both inside and outside the courtroom. The implications for judges, lawyers and psychiatrists that can be drawn from the study are generalisable to other comparable socio-legal frameworks in which mental capacity or competence plays a role and is likely to do so for the foreseeable future.

## 1 Introduction

The concept of mental capacity, as it is called in England and Wales but also sometimes referred to as mental competence, decision-making capacity, etc., is familiar in many jurisdictions, in particular to allow those in the caring professions to answer the question “do I obtain consent from *this* individual?” Most of the late 20th century wave of legal reforms in this area saw an increased focus upon the idea that mental capacity is time- and decision-specific, in other words (1) that anyone, at any time, may lack mental capacity to make a decision (for instance in the immediate aftermath of an accident); and (2) those with permanent impairments of their mind or brain may well be capable of making decisions in relation to one area of their life even if they are not capable of doing so in relation to others. This framework has come to be known as the functional model of capacity.

There is now a sustained challenge, led by the Committee on the Rights of Persons with Disabilities, to the validity of linking mental capacity to legal capacity, the capacity, in particular, to make decisions regarded as having legal effect. The Committee assert, in particular, that mental capacity is not “as commonly presented, an objective, scientific and naturally occurring phenomenon. Mental capacity is contingent on social and political contexts, as are the disciplines, professions and practices which play a dominant role in assessing mental capacity” (Committee on the Rights of Persons with Disabilities, 2014, para 14). Not only does the Committee attack the older models of capacity such as that based on status, i.e. that a diagnosis of an impairment automatically meant that the individual's decisions could not be regarded as legally valid, but also the functional model. The Committee considers that the functional approach to be flawed in part because “it presumes to be able to accurately assess the inner-workings of the human mind and, when the person does not pass the assessment, it then denies him or her a core human right – the right to equal recognition before the law (Committee on the Rights of Persons with Disabilities, 2014, para 15).”

The Committee's challenge to the concept of mental capacity operates at several different levels, some more convincing than others. After all, it is just as possible to assert that a properly implemented legal concept of mental capacity does not deny the individual's rights, but rather responds to situations in which they are unable to make their own decisions: whether – in the case of a person with disabilities – their legal capacity is then respected on an equal basis with others will then depend upon the nature of that response (see further here: Martin, Michalowski, Jütten, & Burch, 2014; Martin et al., 2016; Ruck Keene, 2017).

However, the Committee's challenge does place a particular, and acute, focus on the difficulty of applying the legally ‘neat’ concepts of the functional model of mental capacity across the full complex spectrum of human life. Further, it is undoubtedly the case that there are situations in which determinations of mental capacity are reached which any fair-minded observer would consider to be problematic.

Against this backdrop, a large multi-disciplinary^2^ and multi-jurisdictional^3^, project is underway, one of its aims being to produce educational tools to enable more satisfactory determinations of mental capacity in difficult cases in health and social care contexts. Its first stage has consisted of a detailed review and analysis of the resolution by the Court of Protection in England & Wales of capacity disputes presented to it in the first 10 years of its life, from October 2007 to October 2017.

In focusing this review on cases in which there is an explicit dispute as to the capacity of P, the subject of proceedings, whether between P (or P's family) and the health or social care professionals involved in P's care, or between professionals or expert witnesses called to give evidence, we aimed to shine a spotlight on those cases in which the onus is highest upon the judiciary to apply a statutory formulation of the functional model of capacity so as to achieve a satisfactory resolution of the case before them.

This paper, drawing upon the initial fruits of that review, first outlines how capacity cases came to be in the Court of Protection and examines the role of the Court of Protection in determining capacity. This goes into some detail because (1) many readers will not necessarily be familiar with the workings of the court; (2) there is no readily accessible equivalent overview of the historical developments and the current practice of the court – the closest is Baker J's book (The Hon Mr. Justice Baker, 2018) to which Ruck Keene contributed; and (3) because the court is unusual in being a specialist court charged with the determination of capacity, publishing judgments upon capacity delivered by senior judges, and thus meriting a detailed explanation of the context within which those judgments come to be delivered.

The paper then describes legal and clinical characteristics of disputed capacity cases and asks the question as to how the Court of Protection, now ten years in existence, is doing in complying with its own statutory parameters. The preliminary analysis we present suggests that it is still on a learning curve and has room for improvement. It does, however, highlight the complexity of decision-making capacity in specific contexts, and shows what taking capacity seriously looks like in the court-room, as well as suggesting pointers for future practice.

This is the first in-depth study looking specifically at disputed capacity cases before the Court of Protection. It draws on the largest sample we know of published capacity disputes from a specialist court applying the functional test - the Court of Protection is fairly unique in providing access to such a sample of published judgments on mental capacity. One other author has examined the Court of Protection's case-law through a similar, but narrower lens. In a study of Court of Protection judgments which examined court deference to medical evidence, Paula Case identified 66 judgments in which P's capacity was explored in detail, categorising 12 of these as ‘conflict cases’ in which there was disagreement among experts which the judge needed to resolve (Case, 2016b) - further discussion of this is in Section 5.1. In a prior study examining the role of ‘insight’ in Court of Protection judgments, and particularly in discourses of the court's psychiatric experts, (Case, 2016a), Case found 57 health and welfare judgments, from 2007 to 2015, which discussed capacity in detail, finding that a third of these referenced P's ‘lack of insight’ (Case, 2016a, p. 368) and examining three ‘lack of insight’ cases in detail - all three were dispute cases by our criteria.

For completeness, and by way of comparison, we note that New Zealand has a statutory (functional) capacity test, or to be precise, a series of tests, contained in different parts of the New Zealand Personal and Property Rights Act 1998 [PPPR Act], which is applied by judges of the Family Court and High Court.^4^ Alison Douglass has reviewed Family Court (and some High Court) judgments in which the court's jurisdiction to make an order under the PPPR Act was either contested or discussed, and capacity criteria were directly considered (Douglass, 2016, pp. 181–196, appendix A). She examined reported cases dating from 1988, finding 41 cases meeting her search criteria, but only a subset^5^ of these were defended hearings where capacity was contested or had express discussion of the court's jurisdiction to make an order. She also examined local databases for unreported cases from 2010 to 2015 (Douglass, 2016, pp. 185–186), finding 94 relevant cases – of this group there was a final determination that the subject had capacity in only one case, while in a further 15 cases the court determined that the subject ‘partly’ rather than ‘wholly’ lacked capacity^6^ or the court directed further medical evidence to be obtained. Her study concluded that in depth judicial analysis of whether a person has capacity for purposes of establishing PPPR Act jurisdiction was rare, but she considered that this may be because the court's reasoning is not routinely reported in the judgment, many cases are settled outside court, and outcomes are often unknown for those cases in which the judge orders further medical reports (Douglass, 2016, pp. 194–195).

Therefore, whilst we reach our conclusions on the basis of an analysis of the specialised court in England & Wales, we suggest that they are generalisable to the very many other comparable socio-legal frameworks in which mental capacity/competence plays a role and is likely to do so for the foreseeable future. Indeed, we note that capacity cases from the Court of Protection are now increasingly cited in other jurisdictions.^7^ Further research into disputed capacity cases in other jurisdictions would be useful, not least for comparative purposes.

## 2 Background

### 2.1 The concept of capacity and the Mental Capacity Act 2005

In English law – as in many other legal systems – legal incapacity can arise from a variety of conditions, for instance, being under the age of majority. When it comes to the impact of mental state upon legal capacity, English common law has long had a basic test, to the effect the person concerned had at the relevant time to understand in broad terms what they were doing and the likely effects of their action: a useful overview of the common law in this area as it stood at the start of the reform process in England & Wales can be found in the Law Commission's, 1991 report (Law Commission, 1991, para 2.9–2.42). The courts had also developed specific tests relating to particular forms of decisions with particular consequences, such making a will (*Banks v Goodfellow* (1870) LR 5 QB 549), making a gift (*Re Beaney* (Deceased) [1978] 2 All ER 595), entering into a contract (*Boughton v Knight* (1873) LR 3 PD 64) and conducting litigation (*Masterman-Lister v Brutton & Co and Jewell & Home Counties Dairies* [2003] 3 All ER 162, CA). Alongside these legal developments, clinically-informed concepts of capacity had been developed, in particular to consent to medical treatment, although in a rather more piecemeal way in England & Wales than in other jurisdictions, in particular the United States of America.^8^

Starting in 1989, and in part reflecting broader trends in many Western European and Commonwealth countries,^9^ and in part also a general unease in England and Wales as to whether the common law doctrine of necessity^10^ provided a suitable framework for the delivery of medical treatment to those unable to consent to it, the Law Commission undertook an extensive reform exercise of the law in this area (Law Commission, 1991, Law Commission, 1993, Law Commission, 1995). When it came to the concept of capacity, the project both influenced and was influenced by the important case of *Re C (Refusal of Medical Treatment*) [1994] 1 WLR 290, in which Thorpe J had to decide the definition of capacity which enabled an individual to refuse treatment. He rejected the “minimal competence” test advanced on behalf of the psychiatric patient seeking an injunction to prevent the hospital where he was a patient from amputating his gangrenous leg without his consent, holding that he required more than the capacity to understand in broad terms the nature and effect of the proposed treatment. Rather, he approached matters on the basis that the question was whether the patient's capacity was so reduced by his chronic mental illness that he does not sufficiently understand the nature, purpose and effects of the proffered amputation. Further, following an analysis advanced by the independent psychiatric expert, Nigel Eastman (which was said to reflect that being advanced by the Law Commission^11^), Thorpe J broke down decision-making process into three stages: first, comprehending and retaining treatment information, second, believing it and, third, weighing it in the balance to arrive at choice. Applying that test, Thorpe J found that, even though the patient's “general capacity” was impaired by schizophrenia, “the presumption that [he] has the right of self-determination has not been displaced… I am satisfied that he has understood and retained the relevant treatment information, that in his own way he believes it, and that in the same fashion he has arrived at a clear choice” (*Re C*, para 295).

The process of law reform in England & Wales took over a decade, leading ultimately to the Mental Capacity Act 2005 [MCA 2005].^12^ The Act was intended to establish a comprehensive statutory framework setting out how decisions should be made by and on behalf of adults^13^ whose capacity to make specific decisions is in doubt. Unlike legislation in some other jurisdictions, the MCA 2005 sought to provide a hierarchy of procedures extending from those governing informal day-to-day care and treatment,^14^ to decision-making requiring formal powers and ultimately to court decisions and judgments. The full range of processes was intended to govern the circumstances in which necessary acts of caring can be carried out, and necessary decisions taken, on behalf of those lacking capacity to consent to such acts or make their own decisions.

One significant change that took place between the proposal for legislation by the Law Commission and its final enactment as the MCA 2005 was the introduction, in Section 1, of a set of governing principles. This was very unusual at the time in English legislation,^15^ and reflected in significant part the influence of the Scottish model.^16^ Three of the principles relate to capacity, and two to ‘best interests,’ the touchstone for decision-making when a person lacks capacity to make the relevant decision. The three relating to capacity are that: (1) a person must be assumed to have capacity unless it is established that he lacks capacity; (2) a person is not to be treated as unable to make a decision unless all practicable steps to help him to do so have been taken without success (we examine consideration of this parameter in our study); and (3) a person is not to be treated as unable to make a decision merely because he makes an unwise decision. Section 2(1) MCA 2005 sets out the definition of a person who lacks capacity as follows:

‘For the purposes of this Act, a person lacks capacity in relation to a matter if at the material time he is unable to make a decision for himself in relation to the matter because of an impairment of, or a disturbance in the functioning of, the mind or brain.’

Section 3, in turn, defines what it means to be unable to make a decision. It is a ‘functional’ test, focusing on the ability of the individual concerned to make a particular decision and the processes followed by the person in arriving at the decision – not on the outcome. The definition was expanded from that had been proposed by the Law Commission,^17^ so as to provide that a person is unable to make a decision if he or she is unable (MCA 2005, Section 3(1)):

‘(a) to understand the information relevant to the decision,

(b) to retain that information,

(c) to use or weigh that information as part of the process of making the decision,^18^ or

(d) to communicate his decision (whether by talking, using sign language or any other means).’

During its consultation processes, the Law Commission considered the finely balanced arguments for and against requiring a ‘mental disability’ to be established before someone is deemed to lack capacity (Law Commission, 1993, para 3.10–3.14). The Commission concluded that such a hurdle would serve a useful gate-keeping function, to ensure that decision-making rights were not taken over prematurely or unnecessarily and to make the test of capacity stringent enough *not* to catch large numbers of people who make unusual or unwise decisions. It further, ultimately concluded that the protection offered by a diagnostic threshold outweighed any risk of prejudice or stigma affecting those who need help with decision-making (Law Commission, 1995, para 3.8). When the Commission's proposals were ultimately translated into statute, the term ‘mental disability’ was dispensed with in favour of the wider concept of ‘an impairment of, or a disturbance in the functioning of, the mind or brain’. This covers a wide range of conditions and situations (for instance where a person is unconscious following an accident), and does not equate to (for instance) a recognised diagnosis falling within ICD-11 or DSM-V. The language used in Section 2 is also significant for the phrase “caused by”, indicating that the relationship between the decision-making inability and the impairment of mind or brain must be causal, a concept further developed in case law as the “causative nexus” (*PC v NC v City of York Council* [2013] EWCA Civ 478) and a parameter examined in our study.

The statutory Code of Practice that was produced to accompany the Act suggested that a two-stage procedure had to be applied (Department of Constitutional Affairs, 2007, pp. 44–45):

(1) it must be established that there is an impairment of, or disturbance in the functioning of, the person's mind or brain (the so-called ‘diagnostic test’^19^); and
(2) it must be established that the impairment or disturbance is sufficient to render the person unable to make that particular decision at the relevant time (the ‘functional test’).

The Court of Appeal in *PC v NC v City of York Council*, however, subsequently clarified that the Code of Practice inverted the statutory requirements, and that the functional test should be applied before the so-called diagnostic test. Further, and as also discussed in Section 2.5, despite the use of the term ‘diagnostic’ (which does not appear in the MCA 2005), the courts have made clear that the determination of whether or not a person has the mental capacity to take a particular decision or decisions may well draw upon medical expertise (in particular as to the existence of impairment or disturbance), but is not, ultimately, a ‘medical’ question. Nonetheless, medical – in particular psychiatric – expertise is routinely called upon both within and outside the court setting to determine complex questions of capacity.

### 2.2 The Court of Protection – introduction

The MCA 2005 also created a new specialist court, the Court of Protection (MCA 2005, sections 45 and 47). Not all jurisdictions with developed mental capacity law have created such courts; some have administrative tribunals (for instance in some Canadian jurisdictions and some Australian states), others have left oversight over equivalent legislation to generalist judges (for instance in Scotland). We know of no other jurisdiction which has established a specialist court to apply a statutory version of the functional capacity test.

The tasks of the Court of Protection include:

• to determine whether a person has mental capacity to make specific decisions,
• where the person does lack capacity, to make the decision on their behalf and in their best interests^20^ or to appoint a deputy to do so,
• to make declarations as to the lawfulness of acts done or to be done in relation to a person,
• to determine questions in respect of Lasting and Enduring Powers of Attorney and Advance Decisions to refuse medical treatment;
• (since 1 April 2009), to hear challenges against so-called deprivation of liberty safeguards (‘DOLS’) authorisations.^21^

Although it is a specialist court, not all of its judges sit in the court full-time. There are broadly three tiers of judge who have so-called Court of Protection ‘tickets’;^22^ District Judges, who hear the majority of cases; Circuit Judges, and High Court judges. The Court has a President (who is, conventionally, also the President of the Family Division of the High Court),^23^ and a Vice-President.^24^ There is also the Senior Judge,^25^ who is of Circuit Judge level, and presides over the court's work at its central London base.^26^ Broadly, cases are allocated between tiers depending upon the complexity and gravity of the issues; the most serious cases are reserved for High Court judges sitting in the Court of Protection. Almost all of those judges, at least when it comes to dealing with complex health and welfare cases, are drawn from the Family Division of the High Court, and spend the majority of their time dealing with family cases.

In order to understand the basis upon which the capacity cases analysed below came to be publicly available, and the – relatively small – number of judgments that address issues of capacity, it is necessary to outline both something of the nature of the Court's work, and also when (and why) written judgments come into the public domain.

### 2.3 The Court of Protection – the nature of its work and when applications have to be brought

The majority of the Court of Protection's work consists of deciding upon applications relating to the management of the property and affairs of a person lacking the capacity to do so, often by appointing a deputy to do so on their behalf. The experience of the authors is that the vast majority of these decisions will not be contentious and will be made without a hearing; following changes in 2011, they will often be made by a so-called Authorised Court Officer (an administrative official). These decisions will be very unlikely to result in a written judgment, as the court will simply approve the relevant order put before it. The available statistics (Ministry of Justice)^27^ do not differentiate between contentious and uncontentious applications, nor do they indicate when an application leads to a hearing, but a reasonable estimate is that at least 95% of all the applications would fall into the category of uncontentious applications determined without a hearing.^28^

A very much smaller, but higher profile, part of the Court of Protection's work consists of considering questions of capacity and best interests in the health and welfare context and, related, in the context of considering deprivation of liberty (i.e. compulsory admission to care home or hospital). Of the 241,670 applications made to the Court of Protection between 1st January 2008 and 1st October 2017, 1078 (0.4%) were applications for a one-off ‘personal welfare’ order and a further 8479 (3.5%) were applications relating to deprivation of liberty (Ministry of Justice)^29^.

There are no statutory requirements setting out when a case concerning health and welfare must come to court. As Lady Hale has put it, in the context of the delivery of care and treatment to a person with impaired capacity, the “general authority” given to health care providers provided by MCA 2005, section 5, “*will usually suffice, unless the decision is so serious that the court itself has said it must be taken to court*” (*N v ACCG* [2017] UKSC 22, per Baroness Hale at para 38). Court of Protection judges have also, on occasion, emphasised that in cases of dispute, the onus is on the public body to make application to the court rather than seek to use the mechanisms of the MCA 2005 to stifle dispute;^30^ they have also emphasised that public bodies should seek the sanction of the court before moving adults from their own homes into institutional accommodation, especially in case of dispute.^31^

Health and welfare orders will therefore be sought in a range of situations:

• Sometimes they are for orders permitting medical treatment to be carried out; Certain categories of medical treatment cases have traditionally been brought so as to provide the treating bodies with confirmation that they are acting lawfully,^32^ although the precise basis of the obligation to do so was obscure (Ruck Keene, 2016b) throughout the period under review in the study - the Supreme Court subsequently held in July 2018 that there was, in fact, no legal obligation to do so (*An NHS Trust v Y* [2018] UKSC 46).
• Sometimes they are, in reality, ‘adult care orders’, in other words decisions and declarations relating to the residence and care arrangements and, often, contact arrangements, for an adult with impaired capacity. Such orders have a similar effect to the orders sought by local authorities intervening in the lives of children whose parents are either unable or unwilling to look after them properly. Although such orders are applied for by statutory bodies with caring responsibilities for adults in many different situations, the two largest cohorts are young adults with learning disability (as intellectual disability is known in England and Wales) and elderly persons with dementia (Series, Fennell, Doughty, & Mercer, 2017, pp. 44–45).
• Another class of case which is also brought, usually by a local authority, relate to the intensely personal categories of sex and marriage; these applications are characterised by the focus on the question of capacity: if a person lacks capacity either to consent to sexual relations or to marry, then neither the Court (nor anyone else) can consent on their behalf (see MCA 2005, section 27), so considerations of where the individual's best interests may lie in relation to them are irrelevant.

Separately, but alongside these cases, will be those relating to deprivation of liberty. Unlike the applications noted immediately above, which are normally brought by public bodies, these will often be brought by or on behalf of the person subject to a so-called DOLS authorisation, challenging, usually, whether the deprivation of liberty to which they are subject is in their best interests; challenges are also on occasion brought on the basis that the person, in fact, has the material decision-making capacity, as can be seen in the findings of our study.^33^ This route of access is governed by Section 21A of the MCA 2005 and, as such, these applications are henceforth known as s21A applications.

Applications for one-off welfare orders and those concerning deprivation of liberty are those most likely to raise issues of capacity – and, as detailed in our findings, the majority of our dispute cases pertain (at least in part) to welfare issues. However, it is difficult to estimate how great a focus there will be upon capacity even in this category of cases; the experience of the authors is that, with the exception of those cases concerning sex and marriage, the questions before the court will usually relate not to whether the person has capacity in the relevant domains, but rather to their best interests. Our experience is that the proportion of welfare/deprivation of liberty cases in which there is a contest as to capacity requiring final resolution is much smaller (even if in many cases there may have been initial doubts as to the evidence).

There are limited empirical studies available to offer further clarity on this issue. Series et al. have published a statistical analysis of case files for welfare cases (Series, Fennell, & Doughty, 2017)^34^ active in the Court of Protection 2014/15 but this does not specifically examine whether cases in their sample involved a dispute as to P's capacity. However, their report does comment on cases for which a declaration that P had (rather than lacked) capacity was sought by the applicant and/or made by the judge, which might serve as proxy marker. In the welfare cases examined, they found that the applicants rarely sought a declaration that P had capacity. In fact, this occurred in only 3 out of 153 cases, all relating to medical treatment and all High Court applications (Series, Fennell, Doughty, et al., 2017, p. 38). Moreover there were “very few final declarations that P had mental capacity” (Series, Fennell, & Doughty, 2017, p. 6): 4 cases, all of which involved an application by a public authority seeking a declaration of incapacity (Series, Fennell, & Doughty, 2017, p. 68). However, for the DOLS route, they found that 40% of s21A applications made by P sought a declaration that P had mental capacity. For these s21A applications, there were also many examples of the court finding that P had the relevant mental capacity or that the mental capacity requirement for deprivation of liberty was not met: 8 of 52 completed cases, 15% (Series, Fennell, & Doughty, 2017, pp. 6, 76).

### 2.4 The Court of Protection – publication of judgments

From the outset, the Court of Protection was the subject of fierce criticism in the press as a ‘secret’ court. Not only were judgments hard to access, with the exception of cases concerning serious medical treatment, the hearings themselves were held in private. In part in response to press criticism, and in part in line with a trend towards greater transparency in the family courts, two important developments took place in 2014–5 (see also in this regard: Series, Fennell, Doughty, & Clements, 2015):

• In January 2014, Sir James Munby, the then-President of the Family Division and of the Court of Protection, issued practice guidance citing the “need for greater transparency in order to improve public understanding of the court process and confidence in the court system.” (Sir James Munby, 2014, para 1–2). The guidance was directed at the Senior Judge, nominated Circuit Judges and High Court Judges (Sir James Munby, 2014, para 14), and set out specific categories of cases in which permission should be given for publication of any written judgment produced, absent compelling reasons to the contrary, including, any application for: an order involving the giving or withholding of serious medical treatment; a declaration or order involving a deprivation or possible deprivation of liberty; and an order that P be moved into or out of a residential establishment or other institution (Sir James Munby, 2014, para 17). Such judgments were also to be sent to the website administered by the British and Irish Legal Information Institute (“British and Irish Legal Information Institute,”), known as ‘Bailii’, an important – free – repository of judgments;
• The second development was a move, starting in January 2016, towards a position where almost all attended^35^ hearings are heard in public subject to certain restrictions in relation to the publication of information about the proceedings, in particular the identity of the person concerned and family members.^36^

Even following these changes, however, it is important to understand that a great majority of decisions are made by either Authorised Court Officers (in the context of property and affairs^37^) or District Judges (in the health and welfare context), to whom the guidance set out above does not apply.^38^ It is difficult to be precise about the breakdown, but Series et al.'s study on casefiles in the Court of Protection archives found that of 57 final orders made,^39^ 37 were made by District Judges, 16 by Circuit Judges and 4 by High Court judges (Series, Fennell, & Doughty, 2017, p. 52). This squares with the broader practical experience of the authors.

Further, the guidance of the President did not make it a requirement either that a written judgment be delivered or a transcript be made of a judgment given orally at the end of a hearing. Largely because of pressure of time, many judgments, especially at Circuit Judge level, and even at High Court level, are delivered orally, and unless either a person/body (usually a party) requests – and pays for a transcript – or the court orders a transcript at public expense – there will be no judgment to be made publicly available.

In the circumstances, therefore, the 381 published judgments referred to in the Methods Section, determined by the Court of Protection - that appear on Bailii or on subscription-only databases such as Westlaw or Lexis-Nexis – only relate to a tiny proportion of the applications brought before, or the judgments delivered by, the Court of Protection since 2007. To give a very crude estimate, the statistics show that there were 222,099 orders made by the Court of Protection in the period 1st January 2008 to 1st October 2017 (Ministry of Justice). It is not possible to tell from the statistics whether all of these were final orders in a case made by a judge - in some cases, further, several orders may be made by in the same case over the course of its life; nor is it possible to tell whether all of these orders were accompanied by a judgment, as they may have been orders made by consent which have been simply endorsed by the judge. If one makes the – very generous – assumption that 50% of these orders were final orders made accompanied by a judgment of some form, then we only have 0.34% of the judgments that one might have expected to see.^40^

Finally, and for completeness, we note that, for that small proportion of cases that are appealed from the Court of Protection, there is a strong likelihood, but not a certainty, that the judgment will be published by the Court of Appeal,^41^ and it is certain that any judgment delivered by the Supreme Court will be published. There were 22 Court of Appeal (Civil Division) cases published relating to cases originating in the Court of Protection. There were also 3 cases determined by the Supreme Court in the same period originating from the Court of Protection (none of the Supreme Court cases were concerned with questions of capacity per se).

### 2.5 Capacity before the Court of Protection – Procedure

To set our study in context, it is necessary to give an overview of how the Court of Protection goes about determining whether a person has or lacks capacity to make a specific decision.

Unlike the civil and criminal courts in England, the court's processes:

“are essentially inquisitorial rather than adversarial. In other words, the ambit of the litigation is determined, not by the parties, but by the court, because the function of the court is not to determine in a disinterested way a dispute brought to it by the parties, but rather, to engage in a process of assessing whether an adult is lacking in capacity, and if so, making decisions about his welfare that are in his best interests." (*Cheshire West and Chester Council v P and M* [2011] EWHC 1330 (COP) at para 52, per Baker J)

The court – unsurprisingly – applies the test for capacity set out in section 2 MCA 2005 to the question of whether the person before it has the capacity to make the decision(s) in question; it is also bound by the same principles in section 1, including the principle in s.1(3) that a person is not to be treated as unable to make a decision unless all practicable steps to help him to do so have been taken without success.^42^

In order to apply to the Court of Protection it is necessary to put before the court evidence that the subject of the proceedings, known as ‘P', lacks capacity to take the relevant decision(s). There is a statutory form for this, called a COP3. When the court started, the form could be completed by a registered medical practitioner, psychologist, and in some circumstances, registered therapists such as speech and language or occupational therapists; changes introduced in 2013 widened the pool to include social workers and nurses.

The threshold for engaging the jurisdiction of the court, in section 48 MCA 2005,^43^ is lower than the threshold that the court applies in making a final determination of P's capacity.^44^ Throughout the period under analysis in this paper, the threshold was considered to be that set down in *Re F (Mental Capacity: Interim Jurisdiction) [2009] EWHC B30 (Fam)*, namely whether there was “*sufficient evidence to justify a reasonable belief that P may lack capacity in the relevant regard.*” (*Re F*, para 36).^45^ So long as the threshold is crossed, the court has the power to make interim declarations and decisions, including, importantly, and in discharge of its inquisitorial function, the production of further evidence as to capacity before it makes a final determination of this issue, which it must do so on the balance of probabilities (MCA 2005, section 2(4)). The frequency of expert evidence being obtained and the level of agreement between experts is examined in our study.

In some cases, this evidence will be from a Special Visitor (one of a panel of psychiatrists maintained by the Office of the Public Guardian), who can be directed to produce a report dealing with “such matters relating to P as the court may direct” (MCA 2005, section 49(4)). However, perhaps for historic reasons,^46^ it is far more common in health and welfare cases – especially complex ones – to permit the instruction of an independent expert to report than it is to instruct a Visitor. In line with the inquisitorial nature of the court's jurisdiction the presumption has always been that such experts are instructed jointly between the parties (including, where P is a party, P themselves). This is therefore a very different position to civil litigation in England & Wales where each party may instruct their own expert to advance their case.

The majority of experts instructed in relation to questions of capacity have been psychiatrists (see here: Case, 2016a, Case, 2016b). The experience of the authors is that there has been a very clear trend, however, away from essentially automatic deference by the court to the views of the independent psychiatrist to a more nuanced approach. As Baker J put it in *PH v A Local Authority* [2011] EWHC 1704 (COP), para 16: “[i]*n assessing the question of capacity, the court must consider all the relevant evidence. Clearly, the opinion of an independently-instructed expert will be likely to be of very considerable importance, but in many cases the evidence of other clinicians and professionals who have experience of treating and working with P will be just as important and in some cases more important*,” or as McDonald J put it in *Kings College Hospital NHS Foundation Trust v C & Anon* [2015] EWCOP 80, para 39, “w*hilst the evidence of psychiatrists is likely to be determinative of the issue of whether there is an impairment of the mind for the purposes of s 2(1), the decision as to capacity is a judgment for the court to make*.”

The courts will also routinely hear evidence from health or social care professionals responsible for P, and from P's family or friends. One feature, examined in our study, that often comes as a surprise to those not familiar with the court's work is that the judge will not automatically see the person, nor will the person concerned automatically be a party to the proceedings and represented before the court (see further in this regard: Ruck Keene, Bartlett, & Allen, 2016; Series, Fennell, & Doughty, 2017). However, since 2015, under (now) Rule 1.2 of the Court of Protection Rules 2017, the court must consider as a first step, either on its own initiative or on the application of any person, what measures should be put in place to enable P's participation in the proceedings. These are set down in a ‘menu’ provided by the rule, including P being a party and/or specific provision for P to address (directly or indirectly) the judge.

In practice, although this is not borne out by findings for our sample (perhaps because of the level of judges delivering the judgments), the experience of the authors is that judges do now routinely see and hear from P.^47^ This is especially so in the case of district judges hearing the more routine (and – as above – unreported) cases. It is perhaps not strictly accurate to classify what the judges are gathering from P as “evidence,” 48 although the traditional forensic distinction between sworn and unsworn evidence is now less relevant following changes to the Court of Protection Rules in 2015. 49 The courts have yet to pronounce precisely what it is being done with the information received from P. However, it is clear that on at least some occasions, detailed later in this paper, the court has, in essence, conducted its own assessment of P's capacity in light of how P has presented to the court.^50^

It is important here also to note that there will be occasions when the judge does not hear formally from P in court (with the legal representatives of the other parties present), but goes to see P, usually alone save for the representative of P's litigation friend (the equivalent of a guardian ad litem in some common law jurisdictions).^51^ Judges have been careful to make clear that such visits are not to be used for purposes of gathering evidence, but rather (as in cases involving children), “for the purpose of allowing wishes and feelings to be expressed and to allow [P] to feel part of the proceedings” (*YLA v PM & MZ* [2013] EWHC 3622 (Fam)).^52^ Judges have described the process on such visits as enlightening,^53^ and it is difficult not to gain the impression that, at least on occasion, that enlightenment will be in relation to P's capacity.

A last player who should be highlighted here to understand the context is the Official Solicitor. The Official Solicitor is a statutory office-holder, with a range of functions, most importantly for these purposes being the litigation friend of last resort for P. The Official Solicitor has acted as litigation friend in most of the capacity dispute cases in our study, and, indeed (traditionally) in most of the more complex cases before the Court of Protection; an examination, and critique, of the position can be found in Ruck Keene et al. (2016).

### 2.6 Goals of our study of capacity disputes

Having set the scene, we now turn to a statistical analysis of contested capacity cases before the Court of Protection. Our goals in this analysis are: firstly, to describe the clinical, legal and other factors associated with capacity disputes before the Court of Protection (or Court of Appeal, for those dispute cases appealed from the Court of Protection), and secondly to assess compliance of the judgments with key statutory parameters laid out in 1, 2, 3 of the MCA 2005, as discussed above. The key statutory parameters chosen for examination were:

1. Consideration of practicable steps to maximise P's capacity, a governing principle outlined in section 1 of the MCA 2005.
2. Consideration of the ‘causative nexus’, i.e. reference to the decision-making inability being caused by the impairment of, or disturbance in functioning of, the mind or brain (and not merely citing that an impairment of the mind or brain was present).
3. Engagement with the functional abilities named in section 3 of the MCA 2005, either through citing the relevant functional inability/inabilities in cases for which P is determined to lack capacity or referring to the relevant functional ability/abilities in cases for which P's capacity has been disputed but the judge determines presence of capacity.

These parameters were felt to provide good coverage of the MCA 2005 and therefore adequate tools to assess statutory compliance.

## 3 Methods

### 3.1 Search method

A search was carried out to retrieve all published judgments from Court of Protection, or Court of Appeal (Civil Division) cases on appeal from Court of Protection, determined from 1st October 2007 to 1st October 2017, which were available on Westlaw, LexisNexis and the Bailii databases as of March 2018. This produced a total of 381 Court of Protection judgments^54^ and 22 Court of Appeal (Civil Division) cases.

### 3.2 Contested cases selection criteria

Given the goals of this study, we searched for cases which contained an explicit dispute or contest as to current capacity status of P in relation to a specific issue or issues, in which the dispute was either brought to court for resolution or emerged during proceedings requiring resolution by the judge, and for which resolution occurred with reference to the MCA 2005. This did not include cases in which there was no dispute before the court but the judge undertook a substantive consideration of the individual's capacity (such cases are often, but not exclusively, in the context of serious medical treatment decisions put before the court for endorsement). However, if the judgment documented P to be asserting capacity, in the face of opposition, then this was included as a dispute case even if P's litigation friend (in all cases, the Official Solicitor) conceded this point.

The study's exclusion criteria were as follows:

1. Cases concerning capacity and best interests falling within the study period which were not resolved with reference to the MCA 2005.^55^
2. Cases for which no final declaration was made, including cases for which only an interim declaration under section 48 was made and cases for which no declaration as to P's capacity was made by the judge.
3. Cases pertaining to retrospective judgments of capacity only, for instance as to whether a person had had the capacity at the material time to make a will. In cases pertaining to both current and retrospective judgments of capacity, material related to retrospective judgments was not considered for this study.

There were a total number of 40 cases which met the criteria for this study, including 37 Court of Protection cases and 3 Court of Appeal (Civil Division) cases. These cases are listed in Appendix A.

### 3.3 Coding

Using legal and psychiatric expertise, the team developed a template of categories for extraction of data from the written judgments of the selected cases. The categories evolved via an iterative process of data extraction (see Appendix B for final template). The template focused on key MCA parameters: consideration of practicable steps to maximise capacity, reference to the causative nexus, and engagement of the judge with the four functional abilities of the MCA 2005. It also included relevant legal factors such as modes of participation of P and involvement of expert evidence, clinical factors such as the impairment of mind or brain cited as giving rise to incapacity, and other factors including outcome of the judgment in terms of determination of P's capacity or lack of capacity, and the functional inability cited as relevant to judgments of incapacity. Judgments of selected cases were reviewed by the legal member of the team who carried out the initial extraction of data using the template. This data was subsequently inputted into a statistical package (SPSS), data cleaning was carried out with recoding and adaptation of the template where necessary, and the data was then analysed using descriptive statistics. Fisher exact test was used to explore associations.

## 4 Results

### 4.1 Published dispute cases

**Fig. 1:**
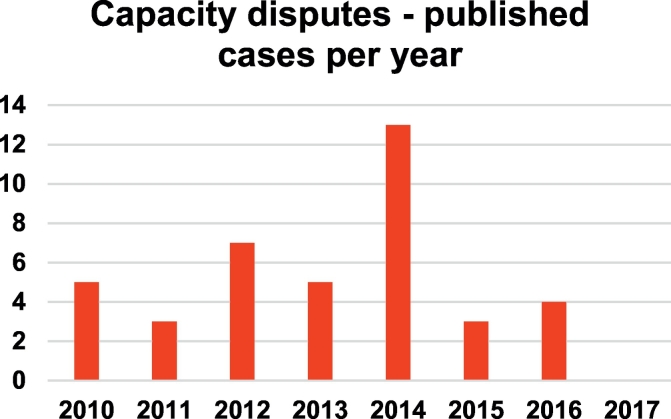
Frequency of published capacity dispute cases, resolved with reference to the MCA 2005, per year from October 2007 to October 2017.

Fig. 1 shows the distribution of the published capacity dispute cases per year. The peak of 13 cases published in 2014 coincided with Sir James Munby's practice guidance relating to publication of judgments, discussed above. There were no published cases pertaining to capacity disputes satisfying our criteria between 1st January 2017 and 1st October 2017.

Regarding level of the judge for the 37 Court of Protection cases, 28 cases (75.7%) were heard before a High Court judge, 2 cases (5.4%) before the Senior Judge, 4 cases (10.8%) before a Circuit Judge, and 3 cases (8.1%) before a District Judge.

### 4.2 Nature of dispute

As shown in Table 1, multiple disputes were frequently at play, and the most common type of dispute was between P and health and/or social care professionals involved in P's care (present in 60% cases).

**Table 1:** Frequency of various court disputes as to P's capacity.

Nature of dispute(s)^a^ as to capacity	N	Percent of cases
Dispute between HSCP^b^ and P	24	60%
Dispute between HSCP and P's family or friends	17	42.5%
Dispute between professionals (HSCP and/or experts)	16	40%
Other dispute	4	10%

a In 16 of 40 cases (40%) there was more than one of these disputes at play.

b HSCP refers to health and/or social care professionals involved in P's care.

### 4.3 Who brought the application?

Table 2 shows that the majority of cases brought to the Court of Protection were from the body with caring responsibility for P. The table is remarkable for the fact that in all cases for which P brought the application there were challenges to DOLS via the section 21A route; this was the route for half of the cases brought by P's family. The ‘other applicant’ category included the Office of Public Guardian in two cases and the beneficiary of gifts made by P in one case. In the fourth case, *A Local Authority v E & Ors* [2012] EWCOP 1639, the application was brought by the local authority, as distinct from the hospital where P was being treated – the local authority bringing the application essentially as a safeguarding matter, and the judge being critical that the health body had not done so, and had not done so some time previously.

**Table 2:** Applicants for disputed capacity cases brought to Court of Protection^a^.

Who brought the application?	N	Percent of cases
Body with caring responsibility for P	22	59.5%
P^b^	7	18.9%
P's family^c^	4	10.8%
Other applicant	4	10.8%

a The three Court of Appeal cases are excluded from this table and considered in the text instead.

b Note that in all 7 cases P brought the application under section 21A of the MCA 2005, challenging the capacity requirement for DOLS.

c In 2 of the 4 cases, P's family member brought the application under section 21A of the MCA 2005.

In addition, three Court of Appeal cases are not included in the table as their procedural history is too complex:

• *RB v Brighton & Hove City Council* [2014] EWCA Civ 561, was originally brought to Court of Protection by P under section 21A of the MCA 2005, and the court's determination that P should be detained in the care home was subsequently appealed by P, but the original decision was upheld.
• In *IM v LM & Ors* [2014] EWCA Civ 37, the application was originally brought by the local authority, and the determination that P had capacity to consent to sexual relations was appealed unsuccessfully by P's mother.
• *PC & NC v City of York Council* was originally brought by the local authority but the determination that P lacked capacity to cohabit with her husband was subsequently appealed successfully by P and her husband.

### 4.4 Participation of P

P was a party to proceedings in all but a small minority of dispute cases (*n* = 37, 92.5%). As a party:

• P was most frequently represented by the Official Solicitor as litigation friend (*n* = 31, 77.5% of all cases).
• P instructed solicitors directly in 4 cases.
• In one case P represented himself.
• There was a single case in which P was named as a party to proceedings with no representation or participation of P.

In 13 cases (32.5%) P spoke directly to the judge, and in another case, P wrote a letter to the judge and this was quoted substantially in the judgment.

• P never spoke directly to the judge when not a party or not represented (4 cases).
• P always spoke directly to the judge when P instructed solicitors or self-represented (5 cases).
• P spoke directly to the judge in 8 of 31 cases for which P was represented by the Official Solicitor.

For our sample, there was no evidence that P was more likely to speak to the judge after 2015, when (as discussed in the Background section) explicit guidance that the court must consider measures to enable P's participation was introduced. We found that P spoke to the judge in 11 of 33 cases (33.3%) before 2015, and in 2 of 7 cases (28.6%) during or after 2015 [Fisher exact test *p* = 1.0].

We hypothesised that P speaking directly to the judge might increase the likelihood of a judgment of P having capacity: when P spoke directly to the judge, P was determined to have capacity for 6 of 12 cases (50%), while when P did not speak to the judge, P was determined to have capacity for 7 of 23 cases (30.4%)^56^ but this difference in proportions was not significant [Fisher exact test, *p* = .29]. Similarly, there was no significant association between the mode of representation of P and a judgment of P having or lacking capacity [Fisher exact test, *p* = .46].

### 4.5 Expert evidence

Evidence on capacity from independent experts (i.e. experts not involved in P's care) was received in 35 of 40 cases (87.5%). In 31, cases expert evidence was received from psychiatrists (alone in 22 cases, with psychologists/social workers in 7 cases, and with a surgeon or gynaecologist in 2 cases) and in 4 cases from psychologists alone.

Did the experts agree among themselves as to P's capacity, and did the judge agree with the experts?

• In 15 of 35 cases (43%), the evidence from experts/professionals disagreed with each other: 4 cases involved disagreement among independent experts only, 10 involved disagreement between independent experts and health/social care professionals involved in P's care, and in 1 case there was disagreement among independent experts and between independent experts and professionals caring for P.
• In 20 of 35 cases (57%), there were no disagreements on P's capacity among the independent experts, or between independent experts and health/social care professionals involved in P's care. In these 20 cases, the judge agreed with the expert view on P's capacity in 16 cases (80%) and opposed the expert view in 4 cases (20% of the time). In all these 4 cases the judge found P to have capacity. While one of the 4 cases was heard before the Court of Appeal, the other 3 cases were heard before the Court of Protection, and in each of these the judge heard directly from P.

One such Court of Protection case was: *WBC v Z* [2016] EWCOP 4, concerning the capacity of a young woman with learning disability and autism spectrum disorder to decide where to live, what care to receive, and what contact to have with others. In his judgment Cobb J concluded that Z had capacity, in doing so diverging from the conclusion of the independent expert and referring explicitly to his own assessment of Z: *“Having read the [expert] reports several times, with care, I was left unsure that I had received a complete or rounded picture of what Z was saying; some of Z's specific responses were included to illustrate the expert opinion that she lacked capacity, but on my reading of them could just as easily have shown merely naivety, immaturity, diffidence, or embarrassment. That naivety, immaturity, diffidence or embarrassment may well not translate into (or necessarily evidence) a lack of capacity. It was, in the circumstances, particularly valuable to have the chance to hear from Z myself, and form an assessment of her, assisted by Dr. Rippon's expertise, when Z herself gave evidence in court at the hearing”*(*WBC v Z*, para 41).

It is also worth noting the following case in which the judge declined to follow the opinion of the psychiatrists giving evidence on behalf of the treating Trust, *King's College Hospital NHS Foundation Trust v C and V* [2015] EWCOP 80, concerning whether a woman had the capacity to decide whether or not to consent to life-saving treatment that her doctors wished to give her following her attempted suicide.^57^ Here the judge gave particular weight to the evidence of C's family on what he termed C's “particular outlook and values” (*King's College Hospital v C,* para 69), and stated clearly, as already quoted in section 2.5, that “*the decision as to capacity is a judgment for the court to make*.” (*King's College Hospital v C*, para 39).

### 4.6 Issues in respect to which capacity was contested

Table 3 shows that in most cases P's capacity was contested in relation to multiple issues. Ten cases pertained to deprivation of liberty and these always involved decisions about care: one case involved a decision on care only, seven involved decisions about care and residence only, while the remaining two involved decisions about care, residence and contact. Two of three marriage cases also dealt with capacity for sexual relations. Medical treatment of physical health included diverse treatment decisions such as: refusal of life-saving dialysis, surgical amputation of a limb, and investigation of rectal bleeding. Medical treatment of obstetric or reproductive health included: decisions about contraception and sterilisation, termination of pregnancy, and delivery by caesarean section. The ‘other issue’ category dealt with a wide range of issues including capacity around educational decisions and a remarkable case on the capacity of P, a woman with dementia who was partly resident in a care home, to decide whether to go on a cruise with her partner: *Cardiff City Council v Ross*, (no neutral citation) Case No. 12063905 (2 November 2011)^58^. Of the 40 cases, only 5 pertained solely to issues outside the welfare domain.

**Table 3:** Frequency of various issues in respect to which capacity was contested.

Nature of issue(s)^a^ in respect to which capacity was contested	N	Percent of cases
Residence	17	42.5%
Care	15	37.5%
Litigation capacity	12	30%
Contact	10	25%
Property and affairs	8	20%
Medical treatment: physical health (excluding obstetric/reproductive)	6	15%
Sexual relations	5	12.5%
Other issue	5	12.5%
Medical treatment: obstetric/reproductive health	4	10%
Power of attorney	3	7.5%
Marriage	3	7.5%
Testamentary capacity	2	5%
Medical treatment: mental health^b^	1	2.5%

a In 27.5% cases (*n* = 11) capacity was contested in relation to a single issue, while in 72.5% cases (*n* = 29) there were multiple issues in respect to which capacity was contested.

b The case pertaining to medical treatment of mental health dealt with treatment of anorexia nervosa including involuntary feeding and is also coded under the medical treatment of physical health category.

### 4.7 Impairment cited as giving rise to lack of capacity

Table 4 shows that learning disability and dementia and related disorders were the most common cited impairments, together accounting for 70% dispute cases. The dementia and related disorders category included two alcohol-related cases (Korsakoff's Syndrome in one case and a possibly alcohol-related dementia in another), two cases of Huntington's disease with cognitive impairment, as well as one case of cognitive impairment said to be due to schizophrenia and ageing. There was no dispute case in which depression was cited as the relevant impairment, and no case in which substance misuse or dependence was cited as giving direct rise to a lack of capacity. In 8 of the 12 cases in which multiple impairments were cited P had a learning disability, and this was comorbid with autism spectrum disorder (in 4 cases), both autism and ADHD (1 case), chronic psychosis (1 case), personality disorder (1 case), and paedophilia (1 case). The other 4 cases with multiple impairments cited involved 2 cases of chronic psychosis and dementia, 1 case of dementia and delirium, and 1 case of acquired brain injury and delirium.

**Table 4:** Frequency of various impairments cited as giving rise to lack of capacity^a^.

Nature of impairment(s)^b^ cited as giving rise to lack of capacity	N	Percent of cases
Learning disability	15	37.5%
Dementia and related disorders	13	32.5%
Chronic psychosis (schizophrenia or schizoaffective disorder)	7	17.5%
Autism spectrum disorder	6	15%
Acquired brain injury	4	10%
Personality disorder	2	5%
Delirium	2	5%
Mood disorder (bipolar disorder with psychosis)	1	2.5%
Eating disorder (anorexia nervosa)	1	2.5%
Other impairment (ADHD, paedophilia)	2	5%

a This refers to impairments cited in all contested cases not merely those for which the judge's final determination was that P lacked capacity.

b In 30% cases (*n* = 12) more than one impairment was cited as giving rise to a lack of capacity, while in 70% cases (*n* = 28) a single relevant impairment was cited.

### 4.8 Judge's determination regarding P's capacity

Table 5 shows that 32.5% of cases were found to have capacity in relation to the issues before the court. In 12.5% cases (*n* = 5), P was found to have capacity in respect to certain issues but lack capacity in relation to other issues before the court. For example, in one case P was found to have testamentary capacity, but to lack capacity to decide regarding retention or sale of a specific property. It is notable that litigation capacity did not always align with capacity regarding the substantive issues considered in court. For example, in one case P was found to have capacity for sexual relations and marriage, but to lack capacity to litigate these issues. In another case, P was found to have capacity for the litigation, but to lack the capacity to decide regarding relinquishing her tenancy agreement and moving to supported accommodation.

**Table 5:** Frequency of judge's determinations regarding P's capacity.

Judge's determinations regarding P's capacity	N	Percent
P lacks capacity	22	55%
P has capacity	13	32.5%
Mixed^a^	5	12.5%

a For the ‘mixed’ group, P was determined to have capacity in respect to certain issues but to lack capacity in relation to other issues before the court.

### 4.9 Functional inabilities

Examining the 23 cases in which P was determined to lack capacity on one or more issues and at least one functional inability was cited by the judge, it is striking that the inability to use or weigh was cited in 21 of 23 cases, and in 7 cases (33%) was the only inability cited. These 7 ‘use or weigh’ cases included Ps with a wide range of impairments – eating disorder, acquired brain injury (two cases), psychosis (alone and with comorbid dementia), learning disability (alone and with comorbid personality disorder). This suggests firstly that ‘use or weigh’ is the inability at the centre of dispute in these contested cases, and secondly, that it is not uncommon to be able to factually understand information while being unable to use or weigh it – these points are discussed further below. The next most commonly cited inability was the inability to understand (15 of 23 cases) but this was cited in isolation in only 2 cases (13%) – both were cases in which P had learning disability (one with comorbid psychosis). The inability to retain information was only cited in conjunction with other functional inabilities and never cited as sole functional inability. This suggests that this criterion does little work, at least in these dispute cases, and supports the hypothesis that without the ability to retain information, it is difficult to understand or use it. In the dispute cases meeting our inclusion criteria there was no case in which an inability to communicate was cited by the judge as a relevant inability.

**Fig. 2:**
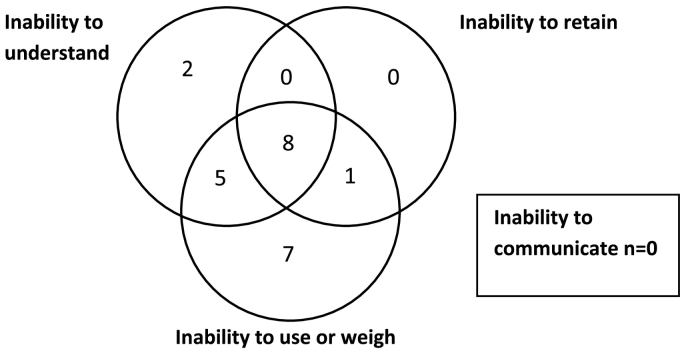
Distribution of functional inabilities cited by judge for P's lack of capacity^1^.

Due to small numbers it was not possible to test associations between specific impairments of the mind or brain and either the judge's determination regarding P's capacity or the functional inabilities cited.

### 4.10 Post-court events

The judgment included an addendum detailing post-court events in only 1 of the 40 cases (2.5%). The case in question pertained to the capacity of a woman to decide regarding a caesarean section. She was found to lack capacity, and the postscript gave details of the safe delivery of her baby via a caesarean section carried out in her best interests: *Re CA* (Natural Delivery or Caesarean Section) [2016] EWCOP 51.^59^

### 4.11 Compliance with key parameters of the MCA 2005

We examined the degree of compliance of the judgments with three key MCA parameters: consideration of the support principle, i.e. whether practical steps were taken to support P to maximise his or her capacity; consideration of the causative nexus, i.e. whether the functional inabilities cited were identified to be *caused by* or *due to* the cited impairment of mind or brain; and finally the engagement of the judge with the four functional abilities of the MCA 2005, namely abilities to understand, retain, use or weigh, and communicate. It was found that the judge:

• considered the support principle in 23 of 40 cases (57.5%)
• considered the causative nexus in 21 of 40 cases (52.5%)
• engaged with at least one of the four functional abilities of the MCA 2005 in 37 of the 40 cases (92.5%). In 1 of the 3 cases in which the judge did not engage with the functional abilities, the judge did however explicitly endorse expert evidence which engaged with the abilities.

For consideration of the causative nexus and engagement with the functional abilities we also examined compliance in the subgroup of 27 cases for which P was found to lack capacity since in these cases the judge might be seen to have a statutory obligation to comply with these two MCA parameters, in order to justify the finding of P lacking capacity. Compliance findings for the subgroup were similar to those for the larger *n* = 40 group, with the causative nexus considered in 15 of 27 cases (55.6%) and the judge citing at least one relevant functional inability in 23 of 27 cases (85.1%).^60^

**Fig. 3:**
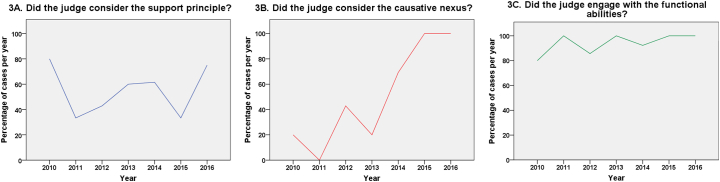
Compliance with key MCA parameters as percentage of cases per year: 3A. Consideration of the support principle, 3B. Consideration of the causative nexus, 3C. Engagement with the functional abilities.

Fig. 3 shows the compliance with key MCA parameters over time. Fig. 3A, which shows compliance with the support principle, does not appear consistent with a trend for improvement over the study period. However, Fig. 3B, showing consideration of the causative nexus, appears consistent with improvement since 2013. While only 4 of 15 cases (26.7%) prior to 2013 considered the causative nexus, 17 of 25 cases (68%) determined in 2013 or after did consider it, with a significant difference between the two groups [Fisher exact test, *p* = .02]. The importance of the causative nexus was highlighted in early 2013 by the Court of Appeal in a case called *PC v NC v City of York Council*, concerning the capacity of a married woman with mild learning difficulties to decide whether to live with her husband upon his discharge from prison. The Court of Appeal emphasised that impairment must be the causal basis of inability, not merely be present or ‘related to’ it. The increase in cases for which the judge considered the causative nexus from 2013 onward^61^ suggests that judges were taking on board the guidance given by the Court of Appeal as to the proper approach to take to the assessment of capacity.

It is difficult to comment on the time trend for Fig. 3C, showing engagement with the functional abilities, as there is near maximum compliance for this parameter. Figures for time trends for the incapacity subgroup (*n* = 27) were similar in form and are therefore not included.

We examined whether legal factors such as P being joined as a party, involvement of the Official Solicitor, and P speaking directly to the judge were associated with increased compliance with the key MCA parameters. We found that if P was a party, the judge was significantly more likely to engage with the functional abilities [Fisher exact test, *p* = .01], and more likely (although this association did not reach significance) to consider the causative nexus [Fisher exact test, *p* = .098]. No other significant associations were found between these legal factors and compliance with the parameters^62^. We also examined the specific association between learning disability cases and compliance with the support principle, hypothesising that judges might be more likely to consider practical steps for a P with learning disability, however there was no significant association here [Fisher exact test, *p* = .512]. Regarding P with an autism spectrum disorder, practical steps were considered in 5 of 6 cases in which P had autism and 18 of 34 cases in which P did not [Fisher exact test, *p* = .216].

### 4.12 Support considered

For the 23 of 40 cases for which the judge considered the support principle, there were:

• 12 cases in which the judge documented previous support given to P, which had been unsuccessful in helping P to achieve capacity for the relevant decision. This included sex education (in 2 cases), sexual offending courses and rehabilitation courses. It also included provision of hearing assistance, use of visual aids and a ‘social story’ around contraception, showing and discussing a DVD of childbirth, P being accompanied and assisted on leave, and health professionals working to gain trust and confidence of P. There was one case (*EM v SC & CM*, [2012] EWHC 1518 (COP)) in which P was accompanied to his home with a member of the RAF (P being a former RAF pilot himself), and letters were requested from his children, to ensure he had the relevant information on options for residence and care. In 3 cases the nature of the support was unspecified.
• 4 cases in which the judge documented previous support given to P which was successful in helping P to achieve capacity for the relevant decision. Support was specified as sex education (in 2 cases), involvement of P in decision-making and giving careful consideration to his religious and cultural beliefs and values, and the careful explanation of information by a trusted professional.
• 4 cases in which potential future support was discussed by the judge or experts. This included ability-appropriate education regarding contraception, “disinterested advice”, memory supports including recording of information in audio or written form, and 1 case in which support was unspecified.
• 2 cases in which the judge criticised the lack of support offered to P and found P to have capacity. In one of these, the judge deemed P had not been provided with relevant information around care options at home.
• 1 case with a passing reference to the need to consider the support principle without further detail.

## 5 Discussion

### 5.1 Main findings

This is the first in-depth study to describe the characteristics of disputed capacity cases before the Court of Protection. While the range of impairments cited as giving rise to incapacity was wide, we found that learning disability and dementia cases together make up 70% of the Court of Protection's work in resolving capacity disputes. This is broadly similar to Series et al.'s study of welfare cases (Series, Fennell, & Doughty, 2017, p. 43)^63^. We found notable gaps in impairments cited– it was remarkable that not a single dispute case pertained to a P with depression, despite the prevalence of this mental disorder in the general population. We found that the Court of Protection ruled on P's capacity for a wide range of issues in contested cases, most commonly residence, care and contact followed by property and affairs, medical treatment for physical health, and sexual relations. This scope fits broadly with data from Series et al.^64^. Within and in addition to these groups, there were many idiosyncratic issues, suggesting that the Court of Protection has an expanding jurisdiction, and that increasing discretion is needed by both experts and judges.

Examining our findings on the judge's determination of P's capacity to make the material decision(s) before them, we can see that, while P was found to lack capacity in a majority of cases, for 13 of 40 cases the judge found that the person, in fact, had capacity. In 5 further cases, the judge found that the person had capacity to make at least some of the decisions in question, even if they lacked capacity to make others. This suggests a nuanced approach taken by the judge with respect to P's capacity, upholding the principle of decision-specificity enshrined in the MCA 2005, and providing good evidence that the court is not operating a status or diagnosis-based approach to capacity.

Indeed, the imperative to avoid a diagnosis-based approach was usefully articulated in *Heart of England NHS Foundation Trust v JB* [2014] EWHC 342 (COP),^65^ concerning the question of whether a woman with paranoid schizophrenia had capacity to consent to or refuse amputation of her gangrenous leg. In this case, Peter Jackson J emphasised that *“the temptation to base a judgment of a person's capacity upon whether they seem to have made a good or bad decision, and in particular on whether they have accepted or rejected medical advice, is absolutely to be avoided. That would be to put the cart before the horse or, expressed another way, to allow the tail of welfare to wag the dog of capacity. Any tendency in this direction risks infringing the rights of that group of persons who, though vulnerable, are capable of making their own decisions. Many who suffer from mental illness are well able to make decisions about their medical treatment, and it is important not to make unjustified assumptions to the contrary”* (*Heart of England NHS Foundation Trust v JB,* para 27).

One of the other cases in which the judge showed clear commitment to fully articulating and applying the functional conception of capacity (and in doing so, overruled expert evidence) was *CC v KK* [2012] EWCOP 2136, concerning the capacity of an elderly woman with dementia to make decisions about her residence and care. In this case Baker J synthesised observations made by other judges in a number of cases, and emphasised that: the roles of the court and the expert are distinct and it is the court that makes the final decision as to the person's functional ability after considering all of the evidence, and not merely the views of the independent expert; professionals and the court must not be unduly influenced by the “protection imperative”; that is, the perceived need to protect the vulnerable adult; the person need only comprehend and weigh the salient details relevant to the decision and not all the peripheral detail. Moreover, different individuals may give different weight to different factors; and capacity assessors should not start with a blank canvas: “*The person under evaluation must be presented with detailed options so that their capacity to weigh up those options can be fairly assessed*” (*CC v KK,* para 68).^66^ These cases illustrate that, even when P has a diagnosed mental disorder, judges may override expert psychiatric opinion on capacity, thus firmly establishing that it is not mental disorder or impairment that is dictating the judgment.

These cases further suggest that, far from blindly following expert evidence, judges do seek to discharge the inquisitorial function of the Court of Protection, in at least some cases. This chimes with Paula Case's study examining deference to medical evidence in the Court of Protection (Case, 2016b)^67^, and it is helpful to turn to her qualitative analysis. In the three cases in which a judge deviated from the expert view, Case finds mixed evidence of the level of actual judicial scrutiny of medical evidence. She notes that a more prominent feature of all cases is that evidence from P is preferred to expert evidence, finding that the judges offer “a normalizing, non-pathological explanation for P's behaviour and style of decision-making based on the judge's own, non-clinical assessment” (Case, 2016b, pp. 186–187). Looking at five cases for which the judge was called on to resolve a dispute between experts, and determined P to have capacity, she found that participation of P (or P's family) facilitated scrutiny of medical evidence in the judgments, although was not always necessary for this scrutiny. In the cases of disagreement between experts for which P was determined to lack capacity, Case comments that at times the judgment appeared to rely on where the “preponderance of expert evidence” (Case, 2016b, p. 192) lay, rather than mapping these assessments to the functional criteria, while in other cases the judge attempted to “‘reconcile’ the two contrasting viewpoints”(Case, 2016b, p. 192), for example by referring to which assessments were lengthiest or more proximal to the hearing, rather than spelling out the conflicting legal norms.

In light of the importance of participation of P in the above cases, our finding that P spoke directly to the judge in less than a third of cases overall is disappointing and suggests an area for development. In some cases, P was said to be unfit to attend the court and this was specifically acknowledged in the judgment, for example in *King's College Hospital v C,* para 58: *“I have not had the opportunity to meet C. She is too ill to attend court and given the need for an urgent decision to be made in this case there has not been time to arrange for me to attend hospital to meet with her.”* However, in other cases it is not clear whether any specific consideration had, in fact, been given to the question of whether P should speak directly to the judge (either at court or by the judge visiting them). In her recent qualitative analysis of participation, based on observation of Court of Protection proceedings and reviewing Court of Protection files, Lindsey argues that P's absence from proceedings is a form of injustice, underpinned by the persistent stereotyping of mentally disabled adults as inherently vulnerable (Lindsey, 2018).

It is also important to highlight the finding that P was applicant to court only for cases pertaining to deprivation of liberty (i.e. via section 21A MCA 2005). This chimes with Series' research looking at welfare cases more generally (Series, Fennell, & Doughty, 2017, p. 6), and is particularly interesting in light of our sample of explicit capacity disputes, a majority of which involved dispute between P and professionals involved in P's care. Notwithstanding the guidance for care bodies to make applications in the event of disagreement, it is legitimate to raise concern about whether subjects of capacity assessment in the community, who wish to challenge their assessment, have meaningful access to mechanisms to do so.

Turning specifically then to our findings on compliance of judgments with three statutory parameters of the MCA 2005, we see that the judge considered the support principle and the causative nexus in over 50% cases respectively and engaged with the named functional abilities (understand, retain, use or weigh, communicate) in over 90% cases, dropping to 85% cases when we looked specifically at the citation of functional inabilities in justifying a determination of incapacity for the relevant decision(s). It is undoubtedly possible to cite this as evidence that the Court of Protection does not always adhere strictly to its own parameters, and we point to this in our section on implications below. However, at this stage it would be remiss not to examine further nuance here. Firstly, we can see in relation to consideration of the causative nexus that a landmark Court of Appeal case, *PC v NC v City of York Council,* appears to show the ability of the system to learn. Secondly, regarding consideration of the support principle, we note at least one Court of Protection case, *LBX v K & Ors* [2013] EWHC 3230 (Fam)*,* that fell outside the scope of our study precisely because the judge considered practicable steps to support P's capacity. The case was excluded as the judge did not make a final declaration as to P's capacity in relation to the issues before the court. The judge of her own motion imposed a temporary halt to proceedings to enable better evidence to be obtained. The evidence she requested was a capacity assessment which used visual aids and other practically supportive techniques, hence giving effect to section 1(3) MCA 2005. These techniques had been used by the independent social worker giving evidence to the court, who had originally instructed primarily to report upon P's best interests but who subsequently raised concerns about whether P in fact had capacity. This example of good practice provides a model for improvement.

Regarding the judge's engagement with the functional abilities, it is interesting that one of the few cases in which the judge did not cite the specific functional inability, in this case the inability which rendered P unable to manage his own affairs and conduct litigation, was in fact an extremely thoughtful and detailed judgment. *A, B and C v X & Z* [2012] EWHC 2400 (COP) was one of the very few cases to consider, implicitly, truly fluctuating capacity, and to take a pragmatic approach to identification of what constitutes ‘the material time’ for purposes of different types of decision, resulting in a decision-specific determination of capacity. Rather, the reasoning relied heavily upon P's decline in ‘executive functioning’, identified to relate to *“complex abstract thinking”* (para 25–26) and not linked to the language of the MCA. In a later case, *NCC v PB and TB* [2014] EWCOP 14, a deficit in executive functioning was tied to the inability to ‘use or weigh’, but the nature of this relation remains unclear.

Finally, an important finding of this study pertains to the work being done by the inability to ‘use or weigh’ in determinations of incapacity in dispute cases. It is remarkable that the inability to retain or communicate were never a deciding factor in resolving the capacity disputes in our sample, suggesting that these concepts are essentially redundant, at least in the Court of Protection setting. Yet ‘use or weigh’ is a newer legal construct and needs more attention in legal and clinical research. In *King's College Hospital v C,* McDonald J gave a useful review of relevant case-law to that date (late 2015), and emphasised that “*a person cannot be considered to be unable to use and weigh information simply on the basis that he or she has applied his or her own values or outlook to that information in making the decision in question and chosen to attach no weight to that information in the decision making process*” (*King's College Hospital v C,* para 38). Often, as in this case, the ‘use or weigh’ ability is questioned in the context of risky or potentially self-harming behaviour on the part of the person, bringing into question the potential for clashes in values^68^ between the person and those in the caring professions - including not just doctors^69^ but also social workers and, in reality, the lawyers and judiciary as well. But the emphasis placed by McDonald J on the values of the person under assessment – and relatedly – the need for the assessor to be aware of their own values, is one that will bear further investigation in the second stage of the work of the current project (see here also: Ruck Keene, 2017).

### 5.2 Study limitations

One potential limitation to our study is that the dispute cases to which we applied scrutiny represent a small proportion of all capacity disputes resolved by the Court of Protection, and that our sample might have characteristics different from those cases for which no published judgment is available. In New Zealand, Douglass encountered a similar limitation in terms of the small proportion of judgments which are published and accessible for research (Douglass, 2016, appendix A, p. 183). Accurately estimating the number of unpublished dispute cases in the Court of Protection would involve obtaining detailed information on all court applications recorded in the available statistics (Ministry of Justice), a pragmatic impossibility. The difficulty is vividly described in the study by Series et al., in which the researchers had to go to significant lengths to reconstruct the life of cases from the individual case-files that they were allowed to study in order to provide an – incomplete - snapshot of the Court's welfare work in 2014–5 (Series, Fennell, & Doughty, 2017).

Nevertheless, regarding representativeness, given that only a minority of cases in our sample (12.5%) pertain to non-welfare issues, it is helpful to compare our data with Series et al.'s data on welfare casefiles. This comparison suggests that our dispute cases are broadly representative of casefiles, or at least those accessible to Series, in terms of a) issues considered by the court, b) impairments of P's mind or brain cited as relevant to the court – as detailed in Section 5.1. This also aligns with a ‘sense check’ by joint first author Alex Ruck Keene, who as a practising barrister has been involved in Court of Protection cases since October 2007.

Furthermore, it is generally the case that, as reflected in the President's guidance set out in the Background section, the more serious the issue before the court, the more likely that the judgment be published, both because of the nature of the subject-matter and because of the likelihood it will have been heard before a judge of the High Court. By virtue of the status of the judge, these judgments have, in practice, both a precedent value (for other judges) and a normative value (for practitioners) that judgments delivered by lower-level judges do not. Our analysis, particularly around implications, sits within the context of this precedent and normative value. Therefore, and especially given the diversity of outcomes of these judgments, our analysis of all 40 published Court of Protection dispute cases is informative and unprecedented.

### 5.3 Implications

For judges, we suggest that our findings have these implications:

• Dispensing ‘inquisitorial’ justice in the Court of Protection is very different to presiding over conventional litigation in common law jurisdictions, the obligations on the judiciary being commensurately higher, including to ensure that both those appearing before them and they, themselves, comply with the – deliberately – onerous obligations of the MCA 2005. One obvious area for development is in relation to the requirement – which applies just as much to the judges of the Court of Protection as it does to any other person applying the MCA 2005 – to consider what practicable steps can be taken to support the person to take their own decision and, where necessary, call a halt to proceedings to require such steps to be taken.
• A clear legal and practical framework should be established for judges to hear directly from P, and should include (1) clarity as to precisely what a judge is doing when they are seeing P; (2) whether specific judicial training might be required; (3) consideration of the starting position that the court goes to P, rather than P being brought to the court, and associated practical guidance to allow this to occur.
• Judges may well wish to consider what tools they require in order to effectively interrogate expert evidence put before them.

For legal practitioners, we suggest that our findings have the following implications:

• There is a need to ensure that any relevant steps that are required before a determination of capacity has been made have been taken. As discussed above, in the English context this includes ensuring that practicable steps to support the individual to take the decision have been taken, and their lack of success (if such be the case) recorded. Otherwise, any actions taken on the basis of an asserted lack of capacity are vulnerable to a retrospective challenge on the basis that relevant practicable steps had not been taken.
• When appearing before the court, there is a need to secure evidence that properly identifies how the relevant statutory test is met. Whilst such evidence may be psychiatric, we have seen above how English courts do not necessarily accept psychiatric evidence (including independent psychiatric evidence) as determinative of the legal question of whether the individual in question has the relevant decision-making capacity.

For psychiatrists, suggested implications are as follows:

• Given the extent to which the principle of decision-specificity is (rightly) upheld in the Court of Protection, psychiatrists preparing evidence for the court should be mindful that working to understand and delineate the relevant decision(s) forms an important pre-condition to assessing the person's capacity.
• Because of the statutory requirement that the decision-making inability be “caused by” the person's impairment of mind or brain, and not merely co-exist with it, psychiatrists should expect to give evidence on this ‘causative nexus.’ This is an area for further research.
• Given that most contested cases hinge on the ‘use or weigh’ ability, psychiatrists should be aware that good quality evidence on this ability is particularly pertinent in court.
• Our findings show that Court of Protection judgments on mental capacity have grown up on particular types of clinical soil - covering some impairments (learning disability, dementia, and, to a lesser extent, psychosis) but not others (notably mood and substance use disorders). This needs to be held in mind when extending the concept of mental capacity, as evolved in the court, to impairments and contexts for which the Court of Protection has less experience.

### 5.4 Next steps

In the second phase of our project, we will draw further upon the dispute cases, as well as a body of over 15 cases, not examined here, in which judges – of their own motion – have undertaken a substantive consideration of the relevant decision-making capacity of P even where there is no dispute. Qualitative analysis will be undertaken on this sample, specifically focusing on the functional abilities of the MCA 2005. We also intend to carry out a comparative examination of the Court of Protection cases and capacity cases in Scotland and New Zealand, jurisdictions where there are similar (but different) capacity frameworks. The next phase of the project, already underway, consists of a series of interviews with practising liaison psychiatrists, lawyers and judges, with a view to collecting and analysing qualitative data on resolution of difficult or contested capacity cases in court and non-court settings. Findings from this combined work will be stress tested with a service user advisory group and used to develop educational tools to bridge the ‘translation’ gap between the legal tests of the MCA 2005 and their application to specific situations in clinical and legal settings.

## 6 Conclusion

Most socio-legal structures will for the foreseeable future continue to depend upon some formulation of mental capacity as an element of legal capacity,^70^ even if, in time, the focus is (as it should be) directed more upon the support that the individual may require to overcome the relevant impairment to exercise their legal capacity. This means that, for the foreseeable future, it will be necessary to have mechanisms to resolve the binary question of whether an individual has or lacks the material mental capacity in any given situation.

Is objectivity, in some relevant sense, possible in mental capacity assessment? Taking a step back from our study's findings and the Court of Protection cases discussed above, and reflecting on work being done in other workstreams of our wider project (Mental Health and Justice, 2018), we wish to draw attention to the importance of asking whether those charged with making determinations of capacity have explained the basis upon which they have reached their conclusion.^71^ Only if they have done so in a fashion which transparently and robustly addresses the relevant statutory criteria can it be said that that the determination is a satisfactory one. Here we have examined how this is done in the Court of Protection, but the onus equally applies to those making capacity determinations outside the courtroom, and it is to those that the next stage of project will be turning.

## Figures and Tables

**Table:**
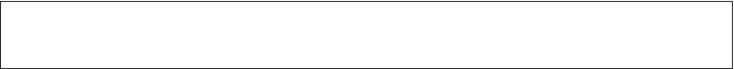
Synopsis of case.

**Table:** A: Nature of issue.

		**Select all that apply**
1	Medical treatment (physical health)	
2	Medical treatment (mental health)	
3	Medical treatment (reproductive and obstetric health)	
4	Residence	
5	Care	
6	Contact	
7	Sexual relations	
8	Marriage	
9	Property and affairs	
10	Power of attorney	
11	Testamentary capacity (statutory will only)	
12	Deprivation of liberty	
13	Litigation capacity	
14	Other	
	**Additional Text**	
	

**Table:** B: Reason for selection.

		**Select all that apply**
1	Dispute about capacity: dispute between health/social care professionals and P	
2	Dispute about capacity: dispute between health/social care professionals and family members/friends	
3	Dispute about capacity: dispute between professionals (health/social care professionals and/or experts)	
4	Other dispute about capacity	
	**Additional Text**	
	

**Table:** C: Pre-court dispute as to capacity?

		**Select all that apply**
1	Dispute about capacity: dispute between health/social care professionals and P	
2	Dispute about capacity: dispute between health/social care professionals and family members/friends	
3	Dispute about capacity: dispute between health/social care professionals	
4	No dispute	
	**Additional Text**	
	

**Table:** D: How matter came to court.

		**Select one**
1	Application by body with caring responsibility for P	
2	Application by family member	
3	Application by P	
4	Other	
	**Additional Text**	
	

**Table:** E: Participation of P.

		**Select all that apply**
1	P a party	
2	P represented by Official Solicitor as litigation friend	
3	P represented by other person as litigation friend	
4	P instructed solicitors directly	
5	P speak directly to the judge	
6	Other	
	**Additional Text**	
	

**Table:** F: Consideration by court of practical steps taken to support P to make the decision(s) in question.

		**Select one**
1	Consideration	
2	No consideration	
**Additional Text**		
	

**Table:** G: Evidence from health/social care professionals involved in care for P.

		**Select one**
1	Evidence received: in agreement	
2	Evidence received: divide between professionals	
3	No evidence received	
	**Additional Text**	
	

**Table:** H: Expert evidence.

		**Select one**
1	Evidence received: expert evidence in agreement	
2	Evidence received: divide in expert evidence	
3	No evidence received	
	**Additional Text (including nature of evidence and names of expert(s)s where applicable)**	
	

**Table:** I: Nature of condition relied upon as giving rise to dispute over/focus on capacity.

		**Select all that apply**	**Text**
1	Chronic psychosis		
2	Mood disorder		
3	Delirium		
4	Eating disorders		
5	Dementia and related disorders		
6	Learning disability		
7	Autism spectrum disorder		
8	Acquired brain injury		
9	Personality disorders		
10	Substance use disorder		
11	Other		
	**Additional Text**		
	

**Table:** J: Functional deficit relied upon as giving rise to incapacity.

		**Select all that apply**	**Text**
1	Inability to understand relevant information		
2	Inability to retain relevant information		
3	Inability to use and weigh relevant information		
4	Inability to communicate decision		
5	No specific deficit relied upon		
6	Not applicable (P found to have capacity)		
	**Additional Text**		
	

**Table:** K: Consideration of ‘causative nexus’?

		**Select one**
1	Consideration	
2	No consideration	
**Additional Text**		
	

**Table:** L: Outcome.

		**Select one**
1	P held to lack capacity	
2	P held to have capacity	
3	Other	
	**Additional Text**	
	

**Table:** M1: Judge's supporting text for ability/abilities found lacking.

		**Select all that apply**	**Text**
1	Ability to understand relevant information		
2	Ability to retain relevant information		
3	Ability to use and weigh relevant information		
4	Ability to communicate decision		
5	No specific ability addressed		
6	Not applicable (P found to have capacity)		

**Table:** M2: Judge's supporting text for ability/abilities found to be present.

		**Select all that apply**	**Text**
1	Ability to understand relevant information		
2	Ability to retain relevant information		
3	Ability to use and weigh relevant information		
4	Ability to communicate decision		
5	No specific ability addressed		
6	Not applicable		

**Table:** N: Evidence endorsed.

		**Select all that apply**	**Text**
1	Ability to understand relevant information		
2	Ability to retain relevant information		
3	Ability to use and weigh relevant information		
4	Ability to communicate decision		
5	No specific ability engaged		
6	No express consideration of evidence		

**Table:** O: Evidence rejected.

		**Select all that apply**	**Text**
1	Ability to understand relevant information		
2	Ability to retain relevant information		
3	Ability to use and weigh relevant information		
4	Ability to communicate decision		
5	No specific ability engaged		
6	No express consideration of evidence		

**Table:**
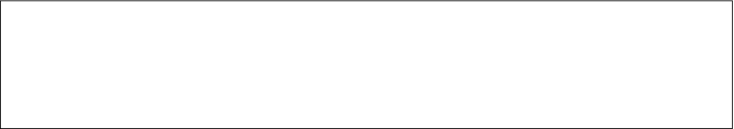
P: Comments and other relevant extracts from judgment.

**Table:** Q: Outcome of events following court's judgment.

		**Select one**	**Text**
1	Recorded in judgment		
2	Another source		
3	Not known		

## Appendix A

### A.1 List of cases included in our study

1. *HBCC v LG & Ors* [2010] EWHC 1527 (Fam)
2. *A Local Authority v Mrs A & Mr A* [2010] EWHC 1549 (Fam)
3. *RT v LT & A Local Authority* [2010] EWHC 1910 (Fam)
4. *LBL v RYJ & VJ* [2010] EWCOP 2665
5. *D v R & S,* [2010] EWCOP 2405
6. *PH v A Local Authority & Ors* [2011] EWCOP 1704
7. *London Borough of Hammersmith and Fulham v MW & JC* 2011 WL 6943174
8. *Cardiff City Council v Ross* (no neutral citation) Case No. 12063905 (2 November 2011)
9. *EM v SC & CM* [2012] EWHC 1518 (COP)
10. *A Local Authority v E & Ors* [2012] EWCOP 1639
11. *Y County Council v ZZ* [2012] EWCOP B34
12. *CC v KK & STCC* [2012] EWCOP 2136
13. *A, B and C v X & Z* [2012] EWHC 2400 (COP)
14. *Re Harcourt: The Public Guardian v A,* [2013] COPLR 69
15. *Re Clarke* [2012] EWCOP 2256
16. *PC & NC v City of York Council* [2013] EWCA Civ 478
17. *Re SB (A patient; capacity to consent to termination)* [2013] EWCOP 1417
18. *A Local Authority v WMA & MA* [2013] EWCOP 2580
19. *A Local Authority v TZ (No 1)* [2013] EWCOP 2322
20. *YLA v PM & MZ* [2013] EWCOP 4020
21. *IM v LM & Ors* [2014] EWCA Civ 37
22. *W NHS Trust v P* [2014] EWHC 119 (COP)
23. *Heart of England NHS Trust v JB* [2014] EWCOP 342
24. *LB Redbridge v G* [2014] EWCOP 485
25. *Re MS* [2014] EWCOP B14
26. *NCC v PB and TB* [2014] EWCOP 14
27. *Wandsworth CCG v IA and TA* [2014] EWCOP 990
28. *RB v Brighton & Hove City Council* [2014] EWCA Civ 561
29. *X v A Local Authority & Anor* [2014] EWCOP 29
30. *GW v A Local Authority* [2014] EWCOP 20
31. *LB Islington v QR* [2014] EWCOP 26
32. *Re CT: The Public Guardian v CT & EY* [2014] EWCOP 51
33. *LB Tower Hamlets v TB & SA* [2014] EWCOP 53
34. *Re DD (No 4: Sterilisation)* [2015] EWCOP 4
35. *King's College Hospital NHS Foundation Trust v C and V* [2015] EWCOP 80
36. *P v Kent County Council* [2015] EWCOP 89
37. *WBC v Z* [2016] EWCOP 4
38. *LB Southwark v KA (Capacity to Marry)*[2016] EWCOP 20
39. *N v A Local Authority* [2016] EWCOP 47
40. *A University Hospital NHS Trust v CA* [2016] EWCOP 51

## Appendix B

### B.1 Contested capacity assessment judgment report template: Court of Protection

Case ID:

Date:

Case Name:

Case citation:

Judge (CoP.

unless otherwise stated):

## Footnotes

1 Note that for 4 of the 27 cases in which P was found to lack capacity for one or more issues, no specific functional inability was cited by the judge. These cases are therefore excluded from Fig. 2

2 The research team consists of an English psychiatrist, a practising English barrister and academic, an American bioethicist/psychiatrist, and a psychiatrist clinical research associate.

3 England, Scotland and New Zealand.

4 “In general terms, the [PPPR] Act says a person lacks capacity if they do not understand the nature or cannot foresee the consequences of decisions, or are unable to communicate them” (Douglass, 2016, p. 181).

5 Full details are not available in her report but of the 41 reported judgments, 6 of 19 made before 2000 were defended hearings where capacity was contested, and there were 10 judgments made from 2001 to 2015 for which jurisdiction was expressly discussed (Douglass, 2016, pp. 184–185).

6 This refers to the fact that, under the PPPR Act, a greater extent of incapacity (‘wholly lacking’) is required to appoint a welfare guardian than, for example, to make a personal order (‘partly lacking’).

7 See, for an Australian example, *PBU v Mental Health Tribunal and Melbourne Health; NJE v Mental Health and Bendigo Health* [2018] VSC 564.

8 There being no English equivalent to the seminal American work of Applebaum and Grisso, starting with their article: (Appelbaum & Grisso, 1988)

9 e.g. In Alberta, Canada, Dependent Adults Act 1978 and Dependent Adults (Amendment) Act 1985; in Victoria, Australia, Guardianship and Administration Board Act 1986; in New Zealand, Protection of Personal and Property Rights Act 1988; in Sweden, Act of 20 December 1974; in France, Loi du 3 janvier 1968; in Austria, Bundesgesetz vom 2 Feber 1983 uber die Sachwalterschaft fur behinderte Personen, Bundesgesetzblatt 1983, 55.

10 In other words, the defence afforded to civil and criminal liability in relation to the delivery of invasive medical treatment to a person reasonably believed to be incapable of consenting to it identified by the House of Lords in in *Re F (Mental Patient: Sterilisation)* [1990] 2 AC 1.

11 In fact, however, the approach then being proposed in the Law Commission's, 1993 report was somewhat different, namely that a person who was able to understand should still be considered to be unable to take a decision if they are prevented by reason of mental disorder from the exercise of “independent will,” (or making a “true choice”) a concept which was intended to “include those who are subject to compulsions arising from their mental disorder itself, [and] those whose mental disorders render them particularly susceptible to the influence of others.” (Law Commission, 1993, para 2.19–2.20). The Law Commission's thinking then evolved, its final position being recorded in the 1995 report (Law Commission, 1995) and discussed below.

12 On the way, the Government undertook further consultation, leading to a policy statement and eventual publication in 2003 of a second Draft Mental Incapacity Bill. The draft Bill was subject to pre-legislative scrutiny by a Joint Parliamentary Select Committee which made a number of recommendations for improvements; what became the MCA 2005 was ultimately introduced to Parliament in 2004.

13 There is, at the time of writing, an open question as to precisely how the MCA 2005 applies in relation to those aged 16 and above. The presumption of mental capacity described below applies from age 16, but the interaction between mental capacity and legal capacity in relation to those 16 to 18 is complex: see *Re D (A Child)* [2017] EWCA Civ 1695.

14 In MCA 2005, section 5, which, in essence, codifies the common law doctrine of necessity.

15 The Mental Health Act 1983, for instance, contained no such principles, although a set of principles is now to be found in the statutory Codes of Practice accompanying the Act in England (Department of Health, 2015) and, separately, Wales (Welsh Government, 2016).

16 Section 1 of the Adults with Incapacity (Scotland) Act 2000 setting out five general principles to govern all ‘interventions’ in the affairs of an adult taken under or in pursuance of the Act.

17 The draft Bill proposed by the Law Commission had proposed a definition that “[f]or the purposes of this Part of this Act a person is at the material time unable to make a decision by reason of mental disability if the disability is such that at the time when the decision needs to be made: (a) he is unable to understand or retain the information relevant to the decision, including information about the reasonably foreseeable consequences of deciding one way or another or of failing to make the decision; or (b) he is unable to make a decision based on that information”. The draft Bill also considered a person to be without capacity if at the material time: “he is unable to communicate his decision on that matter because he is unconscious or for any other reason” (Law Commission, 1995, p. 223).

18 In the report accompanying the draft Bill, and explaining the proposed definition in the previous footnote, the Law Commission had deliberately shied away from this concept. They noted that there were three categories of case where an individual could understand information, but could not use it: (1) because of the effective of a compulsive condition such as anorexia; (2) where their mental disability were unable to exert their will against some stronger person who wishes to influence their decisions or against some force majeure of circumstances; or (3) (the *Re C* situation) where the person is prevented by their disability from being able to believe that information. The Commission continued that: “[c]*ommon to all these cases is the fact that the person's eventual decision is divorced from his or her ability to understand the relevant information. Emphasising that the person must be able to use the information which he or she has successfully understood in the decision-making process deflects the complications of asking whether a person needs to ‘appreciate’ information as well as understand it. A decision based on a compulsion, the overpowering will of a third party or any other inability to act on relevant information as a result of mental disability is not a decision made by a person with decision-making capacity*.” (Law Commission, 1995, para 3.17)

22 In the Court of Protection Rules 2017, these are formally referred to as Tier 1, Tier 2 and Tier 3, but they are conventionally referred to by the categories set out here.

23 Sir Nicholas Wall until 2013, then Sir James Munby, now Sir Andrew McFarlane.

24 Sir Terence Etherton, then Mr. Justice Charles, now Mr. Justice Hayden.

25 Denzil Lush, until his retirement in 2017; now Senior Judge Hilder.

26 The Court of Protection hears cases across the country, although largely for administrative reasons, it was – and in some respects remains - very heavily London-centred.

27 The Ministry of Justice statistics do not cover the period from 1 October 2007 to 1 January 2008, and no statistics appear to be available in relation to this period.

28 Of the applications to the Court of Protection between January 2008 and 1 October 2017, 136,178 were for appointment of a property and affairs deputy; 24,528 for a ‘one-off’ property and affairs order, and 20,926 were applications by an existing deputy or attorney (taken together, representing 75% of the applications); some of these may have been contentious, but only a small fraction. Other categories of applications may well have been predominantly non-contentious, such as 7997 applications for appointment of new trustees; the only categories where disputes are likely are those discussed below for one-off welfare orders and deprivation of liberty cases.

29 The deprivation of liberty statistics are crude, and do not sufficiently differentiate between (1) challenges to administrative authorisations; and (2) applications made by a public body for authority to deprive a person of their liberty (including in situations outside hospitals or care homes).

30 “Significant welfare issues that cannot be resolved by discussion should be placed before the Court of Protection, where decisions can be taken as a matter of urgency where necessary.” *London Borough of Hillingdon v Neary & Anor* [2011] EWCOP 1377, para 33(1) as per Peter Jackson J.

31 See e.g. *Re AG* [2015] EWCOP 78 at para 56 per Sir James Munby, President of the Court of Protection: “Ms Khalique submits, and I am inclined to agree, that the local authority acted unlawfully in removing AG from OG in November 2011 and placing her at HH without having first obtained judicial sanction. Local authorities must seek and obtain appropriate judicial authority before moving an incapacitous adult from their home into other accommodation. Local authorities do not themselves have power to do this. “This is discussed further by Ruck Keene elsewhere (Ruck Keene, 2016a).

33 In the DOLS context, the question is whether the person “lacks capacity in relation to the question whether or not he should be accommodated in the relevant hospital or care home for the purpose of being given the relevant care or treatment” (MCA 2005, Schedule A1, para 14).

34 Series examined 251 Court of Protection files (including application forms, orders, and other court documents) relating to welfare cases (i.e. applications for a one-off welfare order, welfare deputy, or deprivation of liberty review) active in the year 2014–15. Because of considerable access issues, the researchers used a convenience sample which was a hybrid of available ‘complete files’ found in Court of Protection archives at the London Registry (potentially biased to London cases), ‘dummy files’ for active cases which were easier to access but contained less information (these were felt to have decreased likelihood of London-centric sampling bias), and a selection of High Court case files. They used different sub-samples for different questions (Series, Fennell, Doughty, et al., 2017, pp. 34–37).

37 An ACO would never make a final order in any application relating to welfare matters.

38 This is, in part, because the judgments of district judges do not have precedent value, so they represent solely the application of the MCA 2005 to the particular facts of cases before them. That having been said, some judgments of district judges which have been made publicly available have been very influential, in particular those of District Judge Eldergill (see, for instance, his judgment in the case of *Westminster City Council v Sykes* [2014] EWCOP B9.

39 Where the files were complete (see footnote 26 for details).

40 It is helpful to refer again to the study of Series et al. - only 6 of the 251 examined welfare cases had published judgments and these were all High Court cases, although the authors note that some of the High Court cases they examined were potentially ongoing with judgments not yet available (Series, Fennell, Doughty, et al., 2017, p. 67).

41 We cannot state with complete confidence that all judgments delivered by the Court of Appeal will have been published – in particular, there may be some cases in which permission has been refused, either without a hearing (‘on the papers’) or after an oral hearing. In some cases, a written judgment will have been delivered, but this is not inevitable.

42 Both of these flow from the fact it is a statutory court, set up to discharge functions under the MCA 2005.

43 Section 48 of the MCA 2005 relates to ‘Interim orders and directions’, stating that “the court may, pending the determination of an application to it in relation to a person (“P”), make an order or give directions in respect of any matter if (a) there is reason to believe that P lacks capacity in relation to the matter, (b) the matter is one to which its powers under this Act extend, and (c) it is in P's best interests to make the order, or give the directions, without delay.

44 Until 3 October 2017 – i.e. throughout the period under analysis in this paper.

45 Doubt was cast upon this threshold, and whether it was set too low, in *The London Borough of Wandsworth v M & Ors* [2017] EWHC 2435 (Fam).

46 Reflecting the fact that such Visitors were not an option, when equivalent cases were dealt with pre-2007 by the High Court in the exercise of its inherent jurisdiction, and independent experts were instructed instead.

47 ‘Practical guidance’ on facilitating participation of ‘P' and vulnerable persons in Court of Protection proceedings was issued by Charles J, the then-Vice President of the Court of Protection, in November 2016 (Mr Justice Charles, 2016)

48 As Baker J did in *CC v KK and STCC* [2012] EWCOP 2136, where Baker J proceeded (at para 49) on the basis that KK was able to give evidence as to her own capacity which he both could and should weigh up alongside all the other evidence (including that from the experts instructed). This was in some ways an unusual case because KK had been found to have capacity to conduct the proceedings, and it is also clear that KK was cross-examined as to her evidence as to (or possibly, strictly, her assertion of) her own capacity.

49 One of the rule changes in 2015 was to allow (at that point in Court of Protection Rule r85(e)), the court “to admit, accept and act upon such information, whether oral or written, from P […] as the court considers sufficient, although not given on oath and whether or not it would be admissible in a court of law apart from this rule.” (Court of Protection Rules 2017).

50 See e.g. *CC v KK* and also *Re SB (A Patient: Capacity to Consent to Termination)* [2013] EWHC 1417 (COP).

51 See, for example, *Wye Valley NHS Trust v B* [2015] EWCOP 60, concerning whether a man with paranoid schizophrenia had capacity to refuse to undergo the amputation of a severely infected leg (and, if he lacked the capacity) whether it was in his best interests.

52 In this case Parker J noted that “[p]*rior to the hearing commencing before me, I was asked by Ms Giz (*via *email) to see P to ascertain her wishes and feelings: I declined. I was particularly concerned from what Ms Giz wrote that I was being asked to form my own assessment of the strength of her wishes and feelings: and indeed capacity*.”

53 In *A Hospital NHS Trust v CD* [2015] EWCOP 74 (a case concerning the question of whether it was in the best interests of a woman with paranoid schizophrenia to have a total abdominal hysterectomy), Mostyn J observed (at para 31) that it was “*an enlightening experience and one which I would recommend to any judge hearing a similar case* […]. T*he reason it was enlightening for me was that the person I met was different in many respects to the person described in the papers*.”

54 Note, because it took some time for neutral citations to be allocated correctly to Court of Protection cases (and the odd error still occurs), some of these cases had neutral citations suggesting that they were determined in the Family Division.

56 These figures are for the *N* = 35 cases for which there was a determination of either P having or lacking capacity for all issues considered by the court. For the 5 cases in which P was found to have and lack capacity for different decisions, P spoke directly to the judge on one occasion only.

57 An independent jointly instructed psychiatrist considered that P had capacity, but because of “significant shortcomings” in his report (*King's College Hospital v C*, para 52), no weight was placed by the judge upon his conclusions (as opposed to certain factual matters that he had recorded).

58 The judge found that P had capacity, and it appears from media sources that the cruise ship holiday went ahead: (Beckford, 2012)

59 Such a postscript is a common feature of serious medical treatment cases, but a dispute about capacity is rare in the reported serious medical treatment cases.

60 The fact that we looked only at the judge's reliance on functional inabilities rather than reference to abilities present in the *n* = 27 group explains why the number of non-compliant cases increased relative to the *n* = 40 group. There was one case (with a ‘mixed’ or decision-specific outcome i.e. P was found to have and lack capacity for different issues) in which the judge cited functional abilities which were present but not the functional inability relevant to the incapacity finding.

61 Excluding *PC v NC v City of York Council* from the calculation does not affect significance [*p* = .02, Fisher exact test].

62 P being a party was not associated with improved compliance with the support principle [Fisher exact test, *p* = .57]. Involvement of the Official Solicitor was not associated with significantly improved compliance with the support principle [Fisher exact test, *p* = 1.0], causative nexus [Fisher exact test, *p* = .265] or engagement with functional abilities [Fisher exact test, *p* = .121]. P speaking directly to the judge was not associated with improved compliance with the support principle [Fisher exact test, *p* = 1.0], causative nexus [Fisher exact test, p = 1.0] or engagement with functional abilities [Fisher exact test, *p* = .538].

63 Series et al. found that the most common impairments cited as the cause for P's putative incapacity were learning disability (41% cases), dementia (28% cases), mental illness including schizophrenia and depression (22% cases), brain injury (15% cases) and ASD (14% cases) with multiple impairments frequently cited for a single P.

64 When Series et al. looked specifically at applications for declarations of incapacity via personal welfare routes the five most frequently occurring substantive matters were (from most to least frequent): care, residence, contact, treatment of physical illness and sexual relations (Series, Fennell, Doughty, et al., 2017, p. 39).

65 This case deserves highlighting, in particular, not least because of the significant impact that it, as well as *CC v KK* and *King's College Hospital v C*, has subsequently had upon the way in which practitioners, lawyers and the courts apply the Act. This can be seen, in part, from the citation of the relevant case in subsequent published judgments and in part from references to it in guidance for practitioners. Why cases determined at High Court are so influential is, in part, a function of the clarity of the judgment at issue, or the practical utility for training purposes of a turn of phrase used by the judge (a very good example, although relating to best interests, is the observation of Peter Jackson J in *Wye Valley NHS Trust v Mr B* that “a conclusion that a person lacks decision-making capacity is not an ‘off-switch’ for his rights and freedoms”).

66 See also here *Heart of England NHS Foundation Trust v JB*

67 Case identified 66 Court of Protection judgments in which P's capacity was explored in detail, dividing these into 1. ‘consensus cases’ in which there was consensus between experts and the court as to P's capacity (51 cases – 77%), 2. ‘deviation cases’ in which there was no disagreement among experts and the judge opposed the expert view (3 cases – 4.5%), and 3. ‘conflict cases’ in which there was disagreement among experts which the judge needed to resolve (12 cases – 18.2%).

68 Banner argues that there is “inherent normativity” (Banner, 2012, p. 1038) in judgments made about whether a person is using or weighing information to make a decision.

69 This raises the question, of particular interest to psychiatrists, as to the interaction of psychopathology (in C's case, probable personality disorder) and individual values in the decision-making process.

70 Indeed, it is noteworthy that even the most sustained and intellectually rigorous attempts to use the CPRD to escape the gravitational pull of mental capacity appear to circle back to its concepts: see, for instance, (Brosnan & Flynn, 2017) in which the authors come back to a concept of understanding as being at the heart of consent.

71 There are some interesting analogies – as yet unexplored – with the role of rhetoric in judgments about broader medical law matters. See the recent publication by Harrington (Harrington, 2016).

## Footnotes

19 Note that the term ‘diagnostic’ does not appear in the Act. It is in some ways unfortunate that the Code uses this term because it reinforces the medical model of capacity and that only medical practitioners are able to assess it. However, because it is so well-established, the term is used here.

20 ‘Best interests’ in the context of the MCA 2005 is a test that has a strong element of substituted judgment, its purpose being to put the decision-maker in the shoes of the individual, but is ultimately an objective test, where it is possible for a decision to be taken that does not accord with the person's known wishes and feelings. See *Aintree University Hospitals NHS Foundation Trust v James* [2013] UKSC 67, para 23–26, 39, 45, and Ruck Keene and Auckland's paper (Ruck Keene & Auckland, 2015).

21 I.e. administrative authorisations of deprivation of liberty in (i.e. compulsory admissions to hospitals and care homes for those with impaired capacity, under Schedule A1 to the MCA 2005. For an overview of this scheme, which was heavily criticised throughout its life, and is to be replaced in due course, see the Law Commission's, 2017 report (Law Commission, 2017, chapter 4).

32 In particular, decisions to withhold or withdraw artificial nutrition and hydration in a Persistent Vegetative State or a Minimally Conscious State; organ or bone marrow donation by a person who lacks capacity to consent; and non-therapeutic sterilisation (whether of a woman or a man by way of a vasectomy). Until December 2017, these were set out in a Practice Direction to the Court of Protection's governing rules (“Practice Direction E: applications relating to serious medical treatment, supplementing Part 9 of the Court of Protection Rules, 2007,”).

35 I.e. excluding ‘hearings’ which, in fact, consist of a determination by a judge or Authorised Court Officer of an application on the papers.

36 This had, historically, been the position that applied in relation to serious medical treatment applications prior to the coming into force of the MCA 2005.

55 There was a group of cases that started before 1st October 2007 but concluded after 1st October 2007, which were decided under the inherent jurisdiction rather than under the MCA 2005, under transitional arrangements that applied when the Court came into being. These fall outside our study; we also note that most published capacity and best interests cases between 2007 and 2009 were not resolved with reference to the MCA 2005.
